# Working Memory in Navigational and Reaching Spaces in Typically Developing Children at Increasing School Stages

**DOI:** 10.3390/children9111629

**Published:** 2022-10-26

**Authors:** Åsa Bartonek, Cecilia Guariglia, Laura Piccardi

**Affiliations:** 1 Neuropaediatric Unit, Department of Women’s and Children’s Health, Karolinska Institutet, S-171 76 Stockholm, Sweden; 2 Department of Psychology, “Sapienza” University of Rome, 00185 Rome, Italy; 3 Cognitive and Motor Rehabilitation and Neuroimaging Unit, IRCCS Fondazione Santa Lucia, 00179 Rome, Italy; 4 IRCCS San Raffaele, 00163 Rome, Italy

**Keywords:** navigational memory, environmental learning, spatial cognition, life span, large-scale space, small-scale space, children, adolescents, Sweden, external factors

## Abstract

Background: Based on studies of children with motor disabilities on topographic working memory (TWM), no influence of age was reported. The only differences were in the degree of mobility and exploration of the environment. The more active a child was in exploring the environment, the less his/her TWM was poor. However, in typically developing children (TD), exploration of the environment increases with increasing age, and age-related effects have been described. Here, we aim at investigating TWM considering age in TD with the additional question of whether WM in the reaching space differed from that in the navigational space requiring body movements. We hypothesized that WM in both spaces would improve correspondingly with increasing age, assuming that the greater the autonomy in exploring the environment, the better TWM becomes. Method: 120 children (5–16 years old) performed the Corsi Block-Tapping test (CBT) and the Walking Corsi test (WalCT). Results: Statistical analyses evidenced significantly increasing WalCT and CBT spans between each school stage, except in the CBT span between middle stage (MS) and upper stage (US). CBT spans were significantly higher than in the WalCT in the pre-school, lower stage, and MS, with the CBT span increasing until MS, which is sufficient for using spatial orientation strategies effectively. Conclusions: When navigation is gradually controlled, a child may be able to pay increasingly more attention to wayfinding and behavior in traffic. Since the US group even presented as good in the WalCT as young adults living in metropolitan environments, assuming that children may gain spatial orientation from having opportunities to move in their surroundings, this is also relevant for children with motor disabilities.

## 1. Introduction

In studies of topographic working memory (TWM), comparing typical developing children (TD) with children with motor disabilities, difficulties were associated with perceptual-motor conditions in some children, whereas no influence of age was reported in any patient group [1,2]. The present study focuses on the influence of age on working memory (WM) in navigational and reaching spaces in a group of TD. 

The infant’s locomotor experience affects the infant’s autonomy, willfulness, and social cognition [3] making the infant’s memory about how a space is mapped out related to their movements through the space [4]. In a study of spatial memory in children of the ages four to six years, who had either walked independently during the training phase or directed the experimenter while being pushed, performed competently, whereas children who had neither independent locomotor experience nor autonomous choice, whether walking or directing while seated in a pushchair, performed very poorly [5].

Spatial behavior develops gradually during childhood, with landmarks and routes being the minimal elements of spatial representation [6]. The ability to build up a cognitive map of the environment driven by distal cues develops at approximately 7 years of age [7]. Compared to older children and adults using shortcuts when playing a video game, 7- and 8-year-old children were less effective, providing evidence that a distinct developmental change around 9 years of age occurs when children begin to orient and navigate using cognitive maps [8,9]; although relational place strategies, necessary for cognitive mapping, develop even later by the age of 10 [10,11,12]. Indeed, functional Magnetic Resonance Imaging (fMRI) was used to investigate the neurological mechanisms underlying the ability to orient in a virtual interior environment in children aged 10 to 12 years. In comparison to young adults, children were not as proficient at the spatial orientation task and revealed increased neural activity in areas of the brain associated with visuospatial processing and navigation. This finding supported the idea that, as children are maturing in their navigation abilities, they are refining and increasing the proficiency of visuospatial skills for the flexible use of efficient and effective spatial orientation strategies [13].

Spatial cognition is a very high-level skill and it is affected by internal and external factors in the individual. Age [14,15,16] and gender [17,18,19] are two of the many internal factors to influence navigational competence, among external factors such as the complexity of the environment, culture, and the accessibility of reference points, e.g., [20], which are relevant factors and interact with internal factors.

The existence of a WM component devoted to spatial navigation within the visuospatial WM has been supported by several clinical observations of people with a persistent lack of topographical orientation. They were unsuccessful on navigational memory tasks performed in a vista space (i.e., the space that can be visually explored from a single location or with only little eye movements [21]); however, working memory tasks in a space within an arm’s reach were successfully accomplished [22,23,24,25,26,27]. Several studies have also demonstrated that topographic WM, assessed by asking participants to move their body through the space, follows a different trend of development with respect to visuospatial WM tested in the reaching space with participants seated [12,28]. These findings suggest the importance of actively exploring the environment through the whole body versus movements involving solely the arms in the reaching space. From an evolutionary point of view, the two types of actions are different and represent two different reactions to dangers that may be in the environment per se. From a developmental point of view, children become more confident in the very near space, increasing their environmental knowledge when moving through it and exploring it with the whole body. We might also call these two spaces considering the spatial scale in which the actions take place; from this point of view, Hegarty et al. [29] distinguished between small- and large-scale visuospatial tasks and the results they obtain are in line with a partial overlap model of the two scales, which would suggest that some small-scale visuospatial skills are useful at large scales and some are not, with an important distinction related to acted and environmental exploration.

Concerning reaching and navigational space, 4-year-old children performed similarly in working memory measured in reaching and navigational spaces, whereas children from 5 to 6 years of age performed better in the reaching space. This highlights that, as mentioned above, working memory in reaching space increases before topographical memory [12]. With respect to gender, no differences in spatial ability in children have been reported [7,10,11]. Piccardi et al. [28] found no differences in either WM in the reaching or navigational spaces in 4–11 year-old children, whereas, in 6–10 year-old children, girls outperformed boys in the navigational space [12]. The latter finding, however, may have reflected gender-specific cognitive strategies, with girls appearing more focused on the task; thus, the authors recommended these results be taken with caution until confirmed in larger samples of children [12]. The absence of gender differences in developmental age is in line with Newcombe [30], who argues that in the literature, gender differences in spatial abilities of different developmental ages present many conflicting results, which are often related to the way the task is administered rather than the cognitive process itself. Moreover, in children and young adults, 15–25 years of age, no associations with gender differences were found in either working memory in the reaching or navigational spaces [31].

Based on the studies of children with motor disabilities on TWM, no influence of age was reported. This result seems to contradict the studies on typical development, where instead, WM in the reaching space seems to develop earlier. The only major difference that could impact the result obtained in children with motor disabilities is the degree of mobility and exploration that the children have. This finding is of great interest and underscores the importance of the motor system in active exploration that the child has with the environment and in the development of topographic memory itself in early childhood.

The aim of the present study is to investigate TWM in a sample of TD children with the additional question of whether working memory (WM) in a space requiring only arm movements differs from that in a navigational space requiring whole-body movements. Our hypothesis is that WM in both the reaching and navigational spaces will improve corresponding with the increasing age similarly, as in cohorts studied with the same methods in the same country. Indeed, a further question was whether external factors, such as the environment in which children grow up, might influence the narrower aspects of navigation.

In fact, studies conducted on children with motor difficulties and control groups were performed in Sweden, which is a society that favors autonomy for children, who as early as elementary school often travel to school on their own without being accompanied by their parents. This, on the one hand, might explain why age seems to be less important than the ability to explore the environment. Therefore, in this study, the aim is to investigate whether, even in Swedish TD children, age does not affect the development of topographic memory. This would show a differentiation between the memory used in reaching and in vista-navigational spaces, as was apparent in the sample of children of Italian nationality. In fact, in the last three decades in Italy, there has been a progressive separation between people and urban places. Cities are adult-friendly and favor those who travel by car. Children need a reassuring community environment and accessible, safe, and convivial places. The absence of such places produces a detriment to socialization, autonomy, and topographic learning. To this end, the present study aims to investigate the weight of age in children who experience autonomy in moving around the environment earlier than their peers who live in countries with less friendly territory.

To summarize, in the present study we would like to investigate the weight of the internal factor of age on WM in the reaching and vista-navigational spaces, and the internal factor of gender on these abilities in the two spaces. In addition, we would like to evaluate the weight of the external factor of environment/culture on the same abilities in the two spaces.

To this end, we conduct the study using the same method as previous work on Italian children with typical development on a group of Swedish children with typical development, as this allows us to consider the internal factors of age and gender and the external factor of environment/culture.

We expect the external factor environment/culture to have a strong impact on navigational WM development by reducing the difference between WM developmental times in reaching and vista-navigational spaces and thus mitigating the effects of internal factors.

This study sheds light on the weight of cultural differences in the development of WM and provides important insights for the evaluation of these abilities in clinical populations.

## 2. Method

### 2.1. Participants

One hundred and twenty children living in Sweden, fifty-seven males and sixty-three females, mean age = 9.89 ± 3.11, range 5–16 years, were included in this observational study between January 2014 and December 2016. The children were recruited among siblings of patients and through advertisements in a children’s hospital. Criteria for exclusion were the presence of certified cognitive disorders, neurological, or psychiatric disorders. All children had to understand the verbal directives required to accomplish the tests. The study was approved by the Regional Ethical Review Board. Before taking part in the study, printed informed consent was given by the parents and verbal agreement was assured by the children.

The children were grouped according to the Swedish school system in four stages: preschool, which starts the year children turn six (PS), lower stage (grades 1–3) (LS), middle stage (grades 4–6) (MS), and upper stage (grades 7–9) (US) based on the compulsory school system with mandatory attendance between 6/7 and 15/16 years of age (National Agency for Education, 2018 [32]).

### 2.2. Working Memory in the Vista-Navigational and the Reaching Space

All participants performed two WM tests, a visuospatial WM test performed in the reaching space (Corsi Block-Tapping test, CBT) [33] and a topographical WM test (Walking Corsi test, WalCT) [34], administered in randomized order.

**Figure 1:**
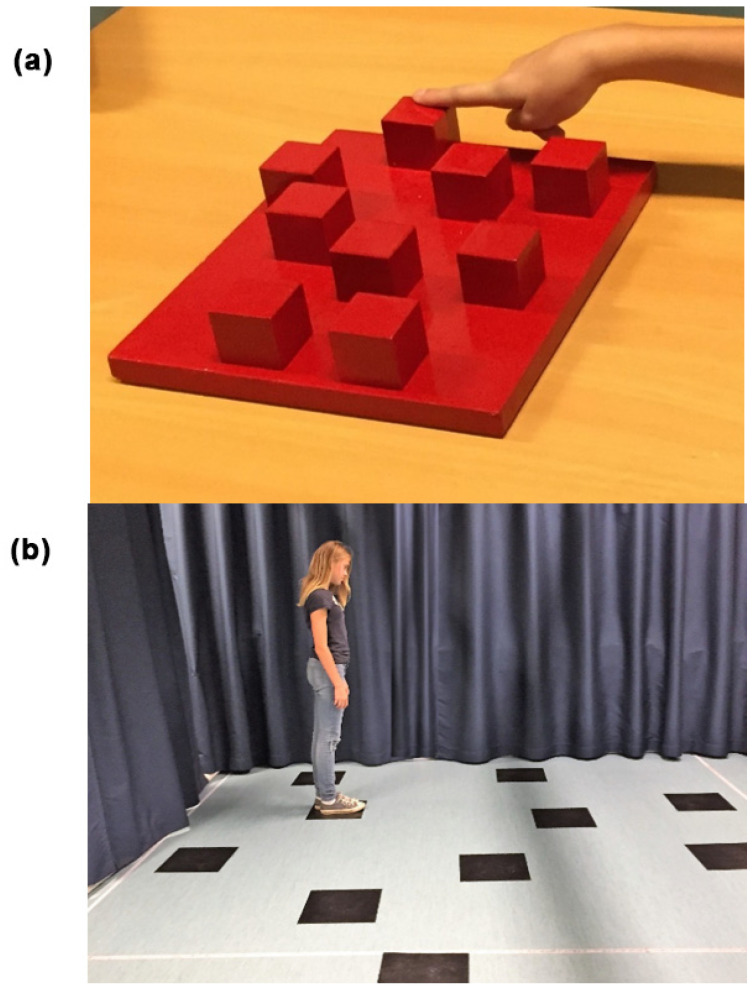
(**a**) The Corsi Block-Tapping test (CBT) consists of nine blocks (4.5 × 4.5 cm) constructed on a baseboard (30 × 25 cm) with numbers observable on the examiner’s side; (**b**) a child performing the Walking Corsi Test (WalCT) on a 300 × 250 cm surface on the floor with nine squares positioned in equal positions as in the CBT.

The methods of both WM tests have been thoroughly described in previous articles [1,2]. The CBT consists of nine blocks fixed on a baseboard (30 × 25 cm) which are numbered on the examiner’s side (Figure 1a). The examiner taps a number of blocks after which the participant taps the sequence in the same order. The test starts with a two-block sequence and increases to a maximum of nine blocks. The test was performed individually in a silent room, with the participant seated on a height-adjustable chair in front of the CBT baseboard opposite the experimenter.

The WalCT allows us to test the TWM in a space that the individual can observe with only little exploratory movements of the head in a so-called “vista space” [21,35], such as single rooms or town squares. The WalCT, consisting of nine squares placed on the floor in equal positions as in the CBT, with a dimension of 300 × 250 cm was arranged in a part of a room encircled by textile draperies (Figure 1b). The examiner demonstrates the sequence by walking on the squares and stopping on each of them for two seconds, increasing from two to nine squares. In both the CBT and WalCT, five trials are performed of which three must be properly completed to continue with a higher sequence. The score corresponds to the longest sequence (span) that was correctly repeated by the participant.

The testing of the CBT and WalCT was performed in Karolinska University Hospital on a single occasion.

### 2.3. Statistics

A Chi-two test was performed to analyze the distribution of gender among the school grades. Pearson correlation coefficients were used to explore associations between age and the two WM tests in the entire group of TD children. An analysis of Variance (ANOVA) was performed to analyze age with respect to school stages and to examine the influence of age on the WalCT and CBT, with a post hoc LSD (least significant difference) test for analysis between the school stages if required. To compare scores of the WalCT and CBT between the school stages, a multivariate analysis of Variance (MANOVA) with a post hoc paired samples test was used. The alpha level chosen for considering a statistical result as significant was *p* < 0.05.

## 3. Results

There was no difference in the distribution of gender among the school stages (*p* = 0.302) (Table 1).

### 3.1. Age with Respect to School Stages

As analysed with the ANOVA, age increased significantly with school stage, F (3,116) = 513.363; *p* ≤ 0.001; partial g2 = 0.930. A post hoc LSD analysis evidenced significantly increasing age between each school stage (Table 1).

### 3.2. Topographical Working Memory in the Vista-Navigational Space

**Figure 2:**
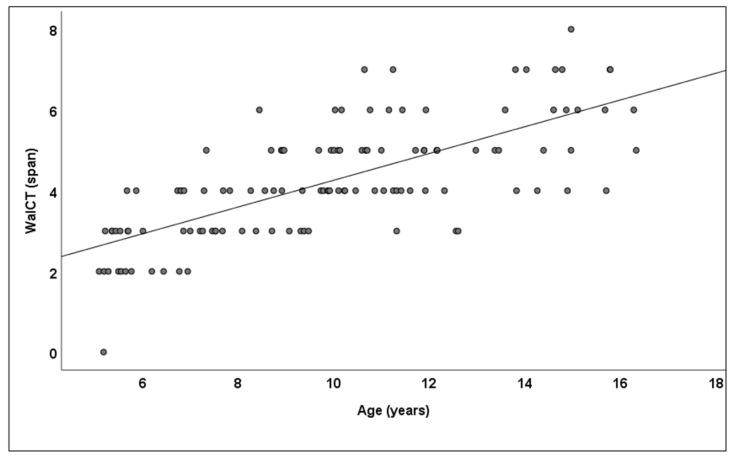
Topographical walking memory with respect to age in the entire study group.

As calculated between age and the entire study group, the Pearson correlation was 0.72 (*p* ≤ 0.001) for the WalCT (Figure 2). Based on an ANOVA, the WalCT score differed significantly between the school stages, F (3,116) = 44.406; *p* ≤ 0.001; partial g2 = 0.535. A post hoc LSD analysis evidenced a significantly increasing WalCT span between each school stage (Table 1). 

### 3.3. Visuospatial Working Memory in the Reaching Space

**Figure 3:**
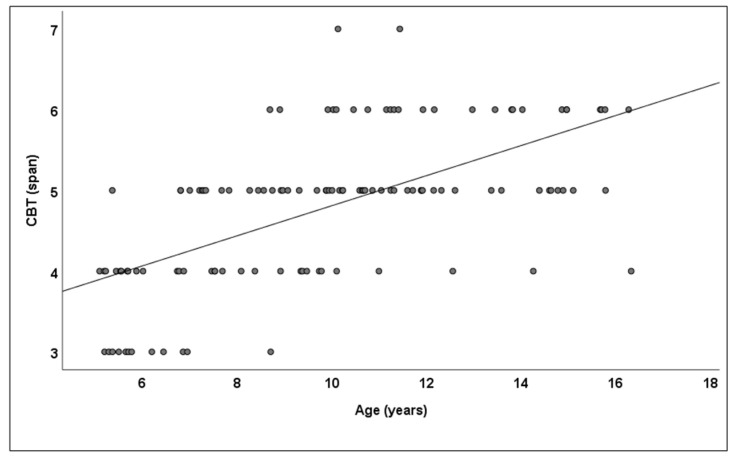
Visuospatial working memory with respect to age in the entire study group.

As calculated between age and the entire study group, the Pearson correlation revealed 0.614 (*p* ≤ 0.001) for the CBT (Figure 3). Based on an ANOVA, the CBT scores differed significantly between the school stages, F (3,116) = 36.403; *p* ≤ 0.001; partial g2 = 0.485. A post hoc LSD analysis evidenced a significantly increasing CBT span between all school stages, except between the MS and US stages (Table 1). 

### 3.4. Working Memory in Vista-Navigational Space Versus Working Memory in Reaching Space

**Figure 4:**
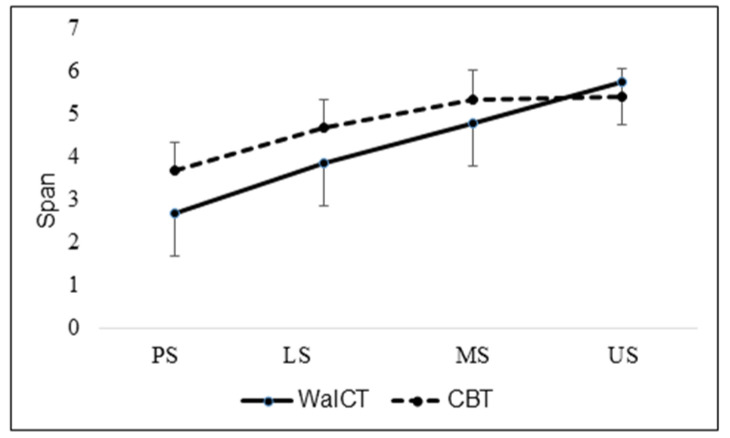
Topographical and visuospatial working memory at various school stages. PS = pre-school (*n* = 27), LS = low stage (*n* = 35), MS = middle stage (*n* = 36), and US = upper stage (*n* = 22).

Based on the MANOVA, the analysis with a post hoc paired samples test evidenced that the spans in the CBT were significantly higher than in the WalCT in school stages PS (*p* ≤ 0.001), LS (*p* ≤ 0.001), and MS (*p* = 0.002) (Figure 4). 

## 4. Discussion

The choice to assess the children’s performance according to stepwise increasing school stages from pre-school to the upper stage (grades 7–9) was based on the assumption that when environmental independence increases, children learn to process topographical stimuli correspondently. The finding of the study confirmed an improvement in TWM with age, which was shown by the strong correlation with a coefficient of 0.7 when analyzing the WalCT score versus age. This is in line with the findings of Lehnung et al. [7] regarding children’s spatial memory and orientation increasing from 5 to 10 years of age, where the 5-year-olds were bound to a cue strategy and the 10-year-olds mastered a place strategy, whereas the 7-year-olds were at an age of transition. By the age of 10, navigational place learning was fully developed [11].

The main finding of the study, and in accordance with our hypothesis, was that the children’s ability to correctly repeat the sequence showed by the examiner, increased in the vista navigational space, continuously with higher school stage. This is in line with the Italian cohorts, [26,27,29], where the results were presented with respect to inclining age groups up to 11 years old. When comparing the results of spatial orientation from the present study with studies using the same method [12,29], the WalCT spans at stages PS, LS, and MS corresponded well with that of Piccardi et al. [12]. Small variations, however, may be explained by the individual variability among children in acquiring the abilities to deal with conflicting frames of reference and to use common coordinate systems [8]. In the US group, aged 13–16 years in the present study, the WalCT score was comparable with data of 50 young adults, 15 to 25 years of age [31]. Based on the results in this study, and with respect to Piccardi et al. [31], it seems that the peak of TWM as measured by the use of the WalCT is reached at approximately 14 years of age. The findings are reasonably in line with Newcombe [8] that adult-level performance and adult patterns of individual differences on cognitive mapping tasks, requiring the integration of vista views of space into environmental space, occur by around 12 years of age.

Furthermore, we wanted to explore the children’s WM in the reaching space. In this vein, we have considered a functional distinction for spaces considering the type of actions individuals performed in the reaching space vs. a vista-navigational space. The space dissociation into reaching space and vista-navigational space is in line with another perspective proposed by Hegarty et al. [29], who distinguish between visual-spatial abilities measured on small and large scales. Their results support the partial dissociation model in which the two sets of abilities rely on some common processes but that ability at each scale of space depends on some unique processes not shared by abilities at the other scale of space. Here, as found in previous studies on children [12,28], we expected WM in the reaching space to increase with age and that WM scores would be higher than in the navigational space. In the Swedish sample, WM in the reaching space increased continuously from the PS to MS groups, however, no improvement was noticed between the MS and US groups; thus, the visual-spatial memory scores stayed persistent from MS at 10 to 12 years of age. This result is mirrored by the moderate correlation with a coefficient of 0.6 when analyzing the CBT score versus age. As for navigational working memory, no available data for comparison of the CBT scores was found in the US group at 13 to 16 years; although, equally as for the navigational task in the same cohort of 15–25 years of age [31], their CBT scores were slightly higher compared with the US group in the present sample. Interestingly, in the cohort of Piccardi et al. [31], the CBT score was significantly negatively correlated with age, showing higher scores in the age group of 15–25 years than in the age group of 26–35 years. Regarding the latter group achieving similar CBT scores to the US group in the present study, this may indicate that visuospatial WM continues to develop in young adults ages. However, according to Murias et al. [13], when children are maturing in their navigation abilities they increase their ability of visuospatial skills for the effective use of spatial orientation strategies. It may therefore be suggested that the development of WM in the reaching space until 12 years of age is sufficient for the effective use of spatial orientation strategies.

It has been reported that 4-year-old children performed similarly well in WM in the reaching and navigational spaces [28], whereas children from 5 to 6 years of age performed better in reaching space [12]. As reported on young adults [31], the visuospatial memory scores were superior to those of the navigational space throughout all age spans. A general explanation for the difference in performances between navigational and reaching spaces could be related to school situations in which children typically learn many spatial and verbal notions in a sitting position, while they become autonomous in exploring unseen spaces later in development. Furthermore, repeating the sequence shown by the examiner in the navigational space means turning the body and thus by changing direction, switching the visual perspective. It, therefore, seems relevant that in the reaching space, where the body position is static omitting any demands of switching perspective, higher scores may be easier to achieve than in the navigational space. Moreover, the reaching space represents the first space in which the individual acts depending on body-object interactions. For example, McKenzie et al. [35] observed that already by 8 months of age, infants perceived that leaning forward extends the range of contact beyond that of reaching alone. Therefore, it is not surprising that WM in reaching space develops before that in navigational space and that children showed a better performance in WM in reaching space than in navigational space. Indeed, reaching space has a key functional role, as it is present in all physical interactions with objects in the environment [36].

In the Swedish sample in the present study, the scores in the reaching space increased with school levels until MS but stayed similar in the US group. On the other hand, the Swedish sample increased their navigational scores with age until the US group at ages 13–16 years, even reaching a higher mean WalCT score than the Italian data of 50 young adults, 15 to 25 years of age. Considering the additional question of the present study to explore the possible influence of external factors on navigation, this finding may be discussed in the light of environmental aspects, where the Swedish children possibly have had benefit from opportunities to move in the surroundings compared to growing up in a metropolitan environment. It may therefore be assumed that there could be external features influencing spatial orientation in the navigational space such as the type of environment in which children grow up. However, the specific research question of how the type of development during growth influences spatial orientation must be further investigated and confirmed. Despite the small participant number that is considered a limitation of the study, the results from the studied population of both types of WM in the reaching as well as navigational spaces seem plausible for not differing substantially from the samples included in the studies by Piccardi and co-workers [12,28,31] using the same methods.

Therefore, we can assume that although external factors (such as the environment in which we live and move) affect our sense of orientation and topographic memory ability, in the case of children with motor difficulties, the difference is more related to aspects of exploratory deprivation that profoundly affects the acquisition of the skill.

Among the different cognitive competencies, to assure success in wayfinding and spatial re-orientation, language, in particular, the comprehension of spatial concepts, seems crucial for navigation as investigated in children [37]. It has also been suggested that preschool spatial experiences may play a central role in children’s mathematical skills around the time of school entry [38] and that spatial visualization and numerical abilities may be related to one another [39]. Moreover, for safe participation in traffic as pedestrians, spatial, psychomotor, and cognitive abilities are insufficient for children below the age of 8 years [40], and the criteria of maintaining spatial awareness were found important when studying safety-relevant aspects of children’s cycling [41]. Interestingly, the efficiency in the processes of spatial cognition and navigational strategies adopted make the difference even in young adults in predicting driving behavior, such as the number of errors, lapses, ordinary and aggressive violations, as well as the number of road-safety behaviors. Indeed, Nori et al. [42] found that drivers using higher navigational strategies are much more able to make correct spatial decisions and travel without incurring violations or fines. These findings in terms of road safety, first as children and later as adults, suggest the importance of deepening in the lifespan and specifically during development of all the processes underlying spatial cognition (i.e., topographic memory, perception, mental imagery, executive functions, and attention).

## 5. Conclusions

In this study, it was shown that WM score in a vista-navigational space increased continuously with age in a Swedish sample, similarly to results found in Italian cohorts using the same methods. The oldest group of 13–16 years even presented as good in the navigational task as an Italian group of 15–25 year-olds, assuming that Swedish children possibly have had benefit from opportunities to move in their surroundings during development more than individuals in an Italian metropolitan environment have. In the reaching space, in the Swedish sample, the working memory score increased until the mean age of 11 years, an age that is close to what has been suggested as sufficient for the effective use of spatial orientation strategies. Furthermore, adult-level performance on cognitive mapping tasks has been suggested to occur by around 12 years of age, which is in line with the results of this study, showing the ability to navigate by controlling body movements in space increasingly, up to the mean age of 14 years of age. This indicates that when navigation in space is gradually controlled, the child is able to pay increasingly more attention to wayfinding and safe participation in traffic. Spatial orientation skills may thus play an important role in the perspective of everyday life in environmental experiences occurring at approximately the end of the middle stage, according to the Swedish school system. Even if the results from the studied population of both working memory in the reaching as well as in the navigational spaces seem plausible, not differing substantially from the Italian sample studied with the same methods, the specific question such as the environment in which children grow up influences spatial orientation, must be confirmed in larger study populations. Studying external factors that may influence the narrower aspects of navigation will help to further distinguish navigation skills from other causes, also highly relevant for children with motor disabilities. Furthermore, the investigation of WM in reaching and vista-navigational space could provide important insights for the rehabilitation of navigational disorders as well as in training skills underlying spatial cognition. Indeed, TWM-based training could enhance this competence in younger children, as was shown by Boccia et al. [43], whereby general navigational skills improved in healthy pre-schoolers after navigational training allowing the acquisition of subsequent steps of human navigation.

## Figures and Tables

**Table 1:** School stages, demographics, mean, standard deviations (SD) (range), age, and spans of WalCT and CBT. PS = pre-school, LS = low stage, MS = middle stage, US = upper stage, f = female, m = male, and ns = non-significant. Significant results are marked in bold text.

School Stage	PS *n* = 27	LS *n* = 35	MS *n* = 36	US *n* = 22	*p*	PS– LS	PS– MS	PS– US	LS– MS	LS– US	MS– US
Grades	pre-school	1–3	4–6	7–9							
Age											
(mean, SD)	5 (0.63)	8.59 (0.93)	11.17 (0.82)	14.77 (0.88)							
(range)	(5.09–6.95)	(7–9.94)	(10.01 –12.97)	(13.37–16.33)	**<0.001**	**<0.001**	**<0.001**	**<0.001**	**<0.001**	**<0.001**	**<0.001**
Gender f/m	14/13	14/21	22/14	13/9	0.308	ns	ns	ns	ns	ns	ns
WalCT span (mean, SD)	2.7 (0.95)	3.86 (0.84)	4.78 (1.01)	5.77 (1.19)	**<0.001**	**<0.001**	**<0.001**	**<0.001**	**<0.001**	**<0.001**	**<0.001**
CBT span (mean, SD)	3.7 (0.66)	4.69 (0.67)	5.33 (0.71)	5.41 (0.66)	**<0.001**	**<0.001**	**<0.001**	**<0.001**	**<0.001**	**<0.001**	0.684

## Data Availability

The datasets generated during and analysed during the current study are available from the corresponding author on reasonable request.
